# Congenital imperforate hymen in a newborn presenting with vaginal protrusion and abdominal distension: a rare case report

**DOI:** 10.1093/jscr/rjag047

**Published:** 2026-02-05

**Authors:** Fatema Almoustafa, Nour Eddin Almustafa, Ahmed Farag, Hamid Al Chamy, Sultaneh Haddad, Sara Jabaly

**Affiliations:** Al Andalus University for Medical Sciences, Faculty of Medicine, Tartus, Syrian Arab Republic; Lattakia University, Faculty of Medicine, Lattakia, Syrian Arab Republic; Kharkiv National Medical University, Faculty of Medicine, Kharkiv, Ukraine; School of Medicine and Medical Sciences, Holy Spirit University of Kaslik, Jounieh, Lebanon; Division of Pediatric, Children's University Hospital, Damascus, Syrian Arab Republic; Damascus University, Faculty of Medicine, Damascus, Syrian Arab Republic

**Keywords:** imperforate hymen, hydrometrocolpos, hydronephrosis, hymenotomy

## Abstract

Imperforate hymen is a rare neonatal anomaly that can cause hydrocolpos and urinary obstruction. We report a newborn girl who presented on day three with abdominal distension and was found to have severe hydrocolpos from an imperforate hymen, leading to bilateral hydronephrosis but with preserved renal function. A hymenal incision effectively drained the accumulated secretions, resulting in rapid clinical improvement and complete resolution on follow-up imaging. Hydrocolpos occurs due to fluid accumulation behind a vaginal obstruction such as an imperforate hymen, and early recognition is essential to prevent complications like hydronephrosis and renal impairment. In this case, timely imaging and focused examination enabled prompt hymenotomy and immediate decompression. This highlights the importance of clinical suspicion, early imaging, and careful genital examination in female neonates. Neonatal imperforate hymen can cause significant hydrometrocolpos and hydronephrosis, emphasizing the need for early recognition and intervention.

## Introduction

An imperforate hymen is a rare congenital anomaly with a worldwide prevalence of 0.014%–0.1%, in which the hymen completely obstructs the vaginal entrance [1–3]. Although often asymptomatic until menstruation between 11 and 15 years of age, it is infrequently detected in the neonatal period. Embryologically, it results from the failure of mesodermal tissue to perforate during late fetal development [1]. In adolescents, the condition usually presents with primary amenorrhea, recurrent abdominal pain, and urinary retention [2, 3]. In neonates, maternal estrogen stimulation can lead to hydrocolpos due to the accumulation of mucoid secretions behind the obstruction, producing a visible vaginal bulge that may compress the urinary tract, causing obstructive uropathy, hydronephrosis, or hydroureter. Early recognition is therefore essential, as delayed diagnosis can result in renal impairment [2, 3]. Diagnosis is typically clinical, supported when needed by transabdominal or transperineal ultrasonography [1]. The primary treatment is hymenotomy under local or general anesthesia to restore normal vaginal outflow [3]. This case is presented to raise awareness of this rare but important cause of neonatal genital swelling and urinary obstruction and to emphasize the need for prompt diagnosis and intervention.

## Case presentation

**Figure 1:**
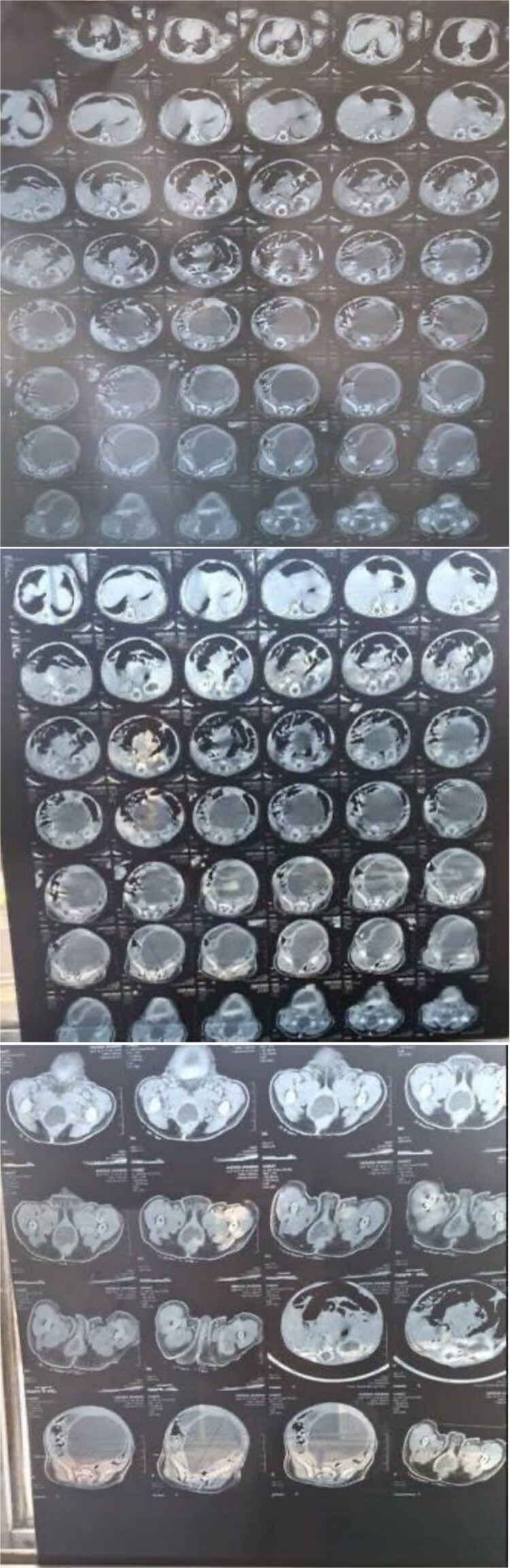
CT demonstrates a markedly distended, fluid-filled vaginal cavity in this infant, consistent with hydrocolpos, extending superiorly and exerting mass effect on adjacent pelvic organs, with secondary bilateral hydronephrosis.

A female infant was born at 38 weeks’ gestation after an unremarkable pregnancy aside from a treated maternal genital infection in the seventh month. Delivery was spontaneous vaginal without complications. Shortly after birth, the family noticed a vaginal bulge but did not seek medical attention. By the third day of life, progressive abdominal enlargement prompted evaluation. A local doctor identified an abdominal mass and ordered a computed tomography (CT) scan (Fig. 1), which showed a large homogeneous abdominopelvic cyst compressing surrounding organs, extending caudally toward the vagina, and associated with marked left hydronephrosis, mild right hydronephrosis, and preserved renal function.

**Figure 2:**
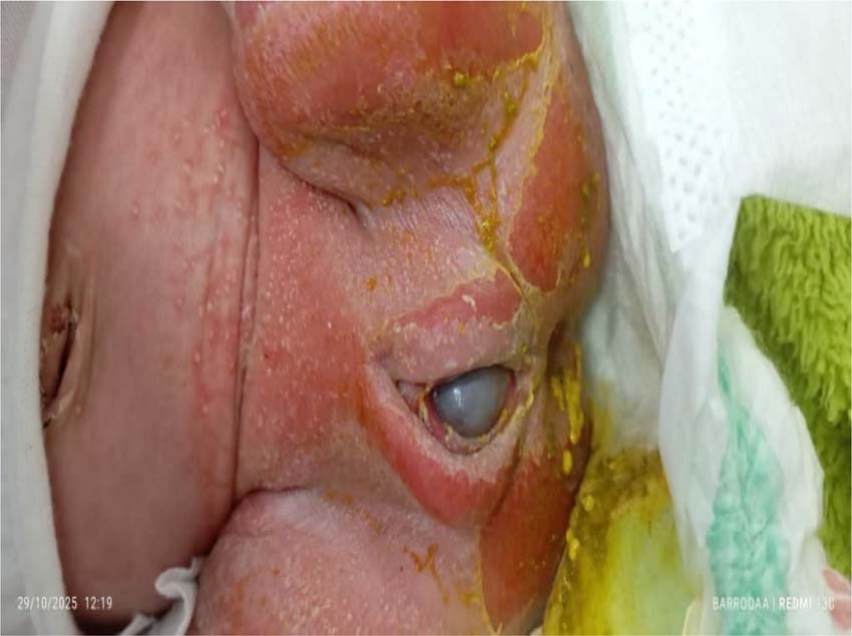
Diaper dermatitis with a visible vaginal bulge.

The differential diagnosis included congenital vaginal cyst, congenital hydrocolpos, congenital uterine outflow obstruction, and congenital malformations of the female genital tract. The infant was admitted to the neonatal intensive care unit. Clinical assessment was consistent with hydrocolpos, and pediatric surgery consultation confirmed the presence of an imperforate hymen with severe hydrocolpos extending above the umbilicus. A hymenal incision was performed, draining a large amount of turbid uterine secretions consistent with hydrocolpos. Postoperatively, abdominal distension resolved, and the hydrocolpos significantly decreased. Other than severe diaper dermatitis (Fig. 2), the infant had no additional clinical concerns.

Laboratory investigations were within normal neonatal ranges, indicating preserved renal and hepatic function despite the hydrocolpos (Table 1). On follow-up, the infant showed clear clinical improvement, and repeat ultrasound confirmed complete resolution of fluid collection after hymenotomy.

**Table 1:** The patient’s laboratory results compared with normal neonatal reference ranges, demonstrating preserved renal and liver function despite hydrocolpos.

Test	Patient value	Normal range (Neonates)	Assessment
WBC (×10^9^/L)	16.5	9–30	Within normal range
Hemoglobin(g/dL)	15	14–24	Within normal range
Platelets (×10^9^/L)	423	150–450	Within normal range
GPT / ALT (U/L)	15	5–45	Within normal range
Urea (mg/dL)	15	5–18	Within normal range
Creatinine (mg/dL)	0.3	0.2–0.8	Within normal range

## Discussion

Hydrocolpos is the enlargement of the vaginal canal caused by accumulation of fluid behind a congenital obstruction. The fluid originates from mucoid secretions produced by uterine and vaginal glands under maternal or neonatal estrogen stimulation. When the volume becomes significant, distention may extend into the uterus, resulting in hydrometrocolpos. Hydrocolpos is rare, occurring in approximately 1 in 16 000 newborn girls and accounting for 15% of abdominal masses in female infants [4]. It results from vaginal blockage combined with hormonally stimulated mucus production. In some infants, a tissue fold may form a one-way valve, creating obstruction similar to certain congenital urethral anomalies. An imperforate hymen is a recognized cause of neonatal vaginal obstruction [5] and was the underlying etiology in this case.

Although usually detected later in life at menarche, neonatal presentation though rare must be recognized. Hydrocolpos may initially be silent, but as fluid accumulates, progressive distention can compress adjacent structures [4]. Such compression may cause obstructive uropathy, hydronephrosis, and hydroureter, and if unrecognized may lead to renal impairment, underscoring the need for early diagnosis [1]. In this case, the infant presented with abdominal distension on day three of life. Imaging demonstrated bilateral hydronephrosis, severe on the left, and a large homogeneous abdominopelvic cyst extending toward the vagina. Timely drainage allowed preservation of renal function.

Hydrocolpos is most often identified prenatally on ultrasound, which typically shows a large fetal abdominopelvic cystic mass containing internal debris. While ultrasound is the primary diagnostic tool, magnetic resonance imaging can further characterize fetal urogenital anomalies. After birth, ultrasound and CT can confirm the diagnosis [6]. In this case, the initial CT produced a broad differential, including congenital vaginal cysts, hydatid cyst, and uterine outflow obstruction. Subsequent targeted examination quickly identified an imperforate hymen causing hydrocolpos.

Hydrocolpos should therefore be included in the differential diagnosis of abdominal distension in female neonates. Early recognition and management are crucial to prevent complications such as progressive hydronephrosis arising from the mass effect of the distended vagina and uterus. Accurate diagnosis is essential before operative intervention. In unstable infants, needle aspiration through the perineum may offer temporary decompression, followed by definitive drainage once the infant stabilizes. In many cases, a catheter may be passed through the obstruction without incision, although surgical intervention is sometimes required [5]. In this patient, the clearly identified imperforate hymen and severity of hydronephrosis warranted immediate hymenotomy. This resulted in rapid clinical improvement following decompression.

## Conclusion

This case demonstrates a rare but significant neonatal presentation of imperforate hymen causing massive hydrometrocolpos and secondary hydronephrosis. Early recognition of the characteristic vaginal bulge, appropriate imaging, and prompt hymenotomy resulted in rapid resolution and preservation of renal function. Genital tract obstruction should always be considered in neonatal abdominal distension, and early intervention is critical to prevent serious complications.

## Clinical takeaways

This case reinforces several important principles.

- Hydrocolpos must be considered in any female neonate with abdominal distension.
- Early imaging and awareness of maternal estrogen–related mucus accumulation are essential for timely diagnosis and prevention of renal damage.
- The rapid improvement after drainage highlights the importance of prompt intervention.
- The identification of the imperforate hymen emphasizes the need for meticulous genital examination in every newborn assessment.

## Highlights

- A newborn female presented with abdominal distension on the third day of life.
- Severe hydrocolpos was identified due to an imperforate hymen.
- Bilateral hydronephrosis was present, but renal function was preserved.
- Hymenotomy was performed, resulting in drainage of secretions and rapid clinical improvement.
